# TAM kinase signaling is indispensable for proper skeletal muscle regeneration in mice

**DOI:** 10.1038/s41419-021-03892-5

**Published:** 2021-06-12

**Authors:** Nour Al-Zaeed, Zsófia Budai, Zsuzsa Szondy, Zsolt Sarang

**Affiliations:** 1 Doctoral School of Molecular Cell and Immune Biology, University of Debrecen, 1 Egyetem square, Debrecen, H-4032 Hungary; 2 Department of Biochemistry and Molecular Biology, Faculty of Medicine, University of Debrecen, 1 Egyetem square, Debrecen, H-4032 Hungary; 3 Dental Biochemistry, Faculty of Dentistry, University of Debrecen, 1 Egyetem square, Debrecen, H-4032 Hungary

**Keywords:** Mechanisms of disease, Immunological disorders

## Abstract

Skeletal muscle regeneration following injury results from the proliferation and differentiation of myogenic stem cells, called satellite cells, located beneath the basal lamina of the muscle fibers. Infiltrating macrophages play an essential role in the process partly by clearing the necrotic cell debris, partly by producing cytokines that guide myogenesis. Infiltrating macrophages are at the beginning pro-inflammatory, but phagocytosis of dead cells induces a phenotypic change to become healing macrophages that regulate inflammation, myoblast fusion and growth, fibrosis, vascularization and return to homeostasis. The TAM receptor kinases Mer and Axl are known efferocytosis receptors in macrophages functioning in tolerogenic or inflammatory conditions, respectively. Here we investigated their involvement in the muscle regeneration process by studying the muscle repair following cardiotoxin-induced injury in Mer^−/−^ mice. We found that Axl was the only TAM kinase receptor expressed on the protein level by skeletal muscle and C2C12 myoblast cells, while Mer was the dominant TAM kinase receptor in the CD45^+^ cells, and its expression significantly increased during repair. Mer ablation did not affect the skeletal muscle weight or structure, but following injury it resulted in a delay in the clearance of necrotic muscle cell debris, in the healing phenotype conversion of macrophages and consequently in a significant delay in the full muscle regeneration. Administration of the TAM kinase inhibitor BMS-777607 to wild type mice mimicked the effect of Mer ablation on the muscle regeneration process, but in addition, it resulted in a long-persisting necrotic area. Finally, in vitro inhibition of TAM kinase signaling in C2C12 myoblasts resulted in decreased viability and in impaired myotube growth. Our work identifies Axl as a survival and growth receptor in the mouse myoblasts, and reveals the contribution of TAM kinase-mediated signaling to the skeletal muscle regeneration both in macrophages and in myoblasts.

## Introduction

Regeneration of the skeletal muscle following injury is an adaptive response of the organ. It involves many stages and the coordinated appearance and action of various cell types [1]. The regeneration process begins with degeneration of myofibers and infiltration of immune cells creating an inflammatory environment [2, 3]. Later, the quiescent muscle stem cells (satellite cells (SCs)) are activated leading to their asymmetric cell division and differentiation into myoblasts that also proliferate and fuse together to form the new myofibers. In the last phase of myogenesis growth of new myofibers, angiogenesis and matrix remodeling take place [4].

During the initial inflammatory phase, neutrophils arrive with the first wave of cells followed by Ly6C^high^ monocytes that differentiate into inflammatory Ly6C^high^ macrophages (MΦs). These cells play a critical role in orchestrating the muscle regeneration partly by phagocytosing the necrotic myofibers and dying neutrophils, partly by releasing various cytokines and growth factors. The initial inflammation creates an environment for the activation, proliferation, and differentiation of SCs, while the resolution of inflammation for the fusion of myoblasts and fiber growth [1, 5, 6]. The timed switch between the two main subsets of MΦs, the Ly6C^high^ ones producing pro-inflammatory cytokines (e.g., tumor necrosis factor-α (TNF-α), interleukin-1β (IL-1β), and interleukin-6 (IL-6)) and the healing Ly6C^low^ ones producing anti-inflammatory cytokines and growth factors (e.g., interleukin-10 (IL-10), transforming growth factor-β (TGF-β), and growth differentiation factor-3 (GDF3)) [6–9], is a key to the proper regeneration process, and it is thought to be induced by the efferocytosis process [10]. Several transcriptional factors, such as Pax7 and MyoD, which regulate the expression of muscle tissue-specific genes (e.g., myosin heavy chain 1 (MYHC1)), and consequently the whole myogenesis [11–13], are under the control of MΦ-derived cytokines that act in autocrine, paracrine, and endocrine manner to orchestrate the immune response as well as the myogenic program of SCs [14, 15].

The TAM receptors (Tyro3/Axl/Mer) form one of the families of tyrosine kinase receptors [16–19]. Mer tyrosine kinase (Mer), a member of this family, is expressed by megakaryocytes, platelets, endothelial cells, epithelial tissue, by the reproductive tissue, and by a broad range of immune cells [20], and is highly upregulated in MΦs during M1–M2 transition [21]. In MΦs, it participates in the GAS6- and protein S-dependent recognition of phosphatidylserine (PS) on the surface of dying cells and thereby facilitates the phagocytic clearance of dead cells, resolution of inflammation, and dampening innate immune responses after acute injury [22–26]. The pathological effects of Mer deficiency were apparent in decreased clearance of apoptotic bodies and in the subsequently increased necrosis in diseases such as atherosclerosis and myocardial infarction [27–30]. Moreover, many in vivo and ex vivo studies demonstrated that Mer-deficient monocytes and MΦs display increased inflammatory phenotype, as following activation, they produce larger amounts of pro-inflammatory and decreased amounts of anti-inflammatory cytokines [23, 25, 29]. In this study, we tested the hypothesis that by promoting efferocytosis and by regulating cytokine production MΦ, Mer might be involved in the regeneration of injured muscle.

## Materials and methods

### Reagents

All reagents were obtained from Sigma-Aldrich (Budapest, Hungary), except when indicated otherwise.

### Experimental animals

Experiments were carried out using 2–4-month-old full-body knockout Mer^+/+^ and Mer^−/−^ males and where it is indicated female mice. Mice were purchased from the Jackson Laboratory (Bar Harbor, Maine, USA) and bred in heterozygous form under specific pathogen-free conditions in the central animal facility of the University of Debrecen. All animal experiments were approved by the Animal Care and Use Committee of the University of Debrecen (DEMÁB) with a permission number 7/2016/DEMÁB.

### Cardiotoxin-induced muscle injury model

Mice were anesthetized by intraperitoneal injection of pentobarbital (80 mg/kg mouse). After anesthesia, muscle injury was induced by injecting 50 μl of 12 μM cardiotoxin (CTX) (Latoxan, Valence, France) in phosphate-buffered saline (PBS) into the tibialis anterior (TA) muscle. The size of the control and treated groups was the same as reported by others in similar experiments [31]. There were no inclusion or exclusion criteria used in the selection of the animals. Animals from each cage were randomly allocated to the control or treated groups, but no blinding was used. Mice were sacrificed and muscles were harvested at various time points following injury. Samples were frozen for immunohistochemical staining or processed for Western blot analysis, cell, or mRNA isolation. In some experiments, 10 mg/kg body weight pan-TAM tyrosine kinase inhibitor BMS-777607 was injected intraperitoneally into wild-type mice on the first and third, or on the fifth, seventh, and ninth day of cardiotoxin injury.

### Isolation of muscle-derived CD45^+^ leukocytes and F4/80^+^ MΦs

CD45^+^ leukocytes or F4/80^+^ MΦs were isolated from TA muscles using a modified protocol developed by Patsalos et al. [31]. For the phagocytosis experiments, muscle-derived MΦs were suspended in Dulbecco’s modified Eagle’s medium (DMEM) supplemented with 10% fetal bovine serum (FBS) (Invitrogen, Carlsbad, USA), penicillin (100 units/ml; Invitrogen, Carlsbad, USA), streptomycin (100 mg/ml; Invitrogen, Carlsbad, USA), and 2mM L-glutamine and incubated in 12-well plates (3 × 10^5^ cells/well) for 48 h at 37 °C. After two days, floating cells were washed away, and fresh medium was added to the attached cells for an additional 24 h.

### Quantification of intramuscular immune cells by flow cytometry

The magnetically separated muscle-derived CD45^+^ cells were stained with a combination of Alexa Fluor 488-conjugated anti-F4/80 antibody (MF48020, Invitrogen, Carlsbad, USA) and Alexa Fluor 647 conjugated anti-Ly6G/Ly6C (GR-1) antibodies (108418, BioLegend, San Diego, USA) at room temperature for 15 minutes. Cells were gated based on their forward- and side-scatter characteristics. Macrophages were gated as GR-1 negative and F4/80 positive, while neutrophils as F4/80-negative and GR-1-positive cells. F4/80-positive macrophages were also analyzed for Ly6C, CD206 or MHCII expressions following staining with Ly6C PerCP-Cy5.5 (128012, BioLegend, San Diego, USA), CD206-PE (141705, BioLegend, San Diego, USA) or MHCII-FITC (107605, BioLegend, San Diego, USA) antibodies, respectively. Fluorescent intensity was detected with a Becton Dickinson FACSCalibur instrument.

### Cell sorting

The magnetically separated muscle-derived CD45^+^ cells were stained with a combination of Alexa Fluor 488-conjugated anti-F4/80 antibody and Ly6C PerCP-Cy5.5 antibodies at room temperature for 15 minutes. Macrophages were gated as F4/80 positive cells and further separated Ly6C^high^ and Ly6C^low^ populations based on their Ly6C expression level on BD FACSAria™ III Cell Sorter.

### Immunofluorescent staining and immunohistochemistry

Muscles from control mice or at 2-, 4-, 10- or 22-day post injury were dissected for histological assessment. Muscles were snap-frozen in liquid nitrogen-cooled isopentane and kept at −80 °C. About 7-μm cryosections were cut at −20 °C using a 2800 Frigocut microtome (Leica, St Jouarre, France) and were kept at −20 °C until further analysis. Hematoxylin and eosin (H&E) staining was performed to assess the overall morphology and the presence of necrotic fibers following injury. To calculate the cross-sectional and collagen-stained areas, briefly frozen muscle sections were incubated 10 mM citric acid–sodium citrate buffer (pH 6.0) for 15 min, then in blocking solution (50% FBS in PBS) for 1 h at room temperature followed by the incubation of the muscle sections with Dylight 488-conjugated anti-laminin B (PA5-22901,Invitrogen, Carlsbad, USA) (1:100), or anti-collagen 1 antibody (SAB4500362) (1:100) at 4 °C overnight followed by Alexa Fluor 488-conjugated goat anti-rabbit IgG secondary antibody. Slides were counterstained with 4 μg/ml 4′,6-diamidino-2-phenylindole (DAPI) (Invitrogen, Carlsbad, USA). Images were analyzed using ImageJ software (National Institutes of Health, Bethesda, USA) with muscle morphometry plugin. Areas with fibers containing centrally-located nuclei were considered as regenerating muscle parts. CSAs are reported in μm^2^, while the amount of collagen deposition as percent of the total examined regenerating area. For MYHC4 staining C2C12 cells were fixed with ice-cold methanol and washed three times with PBS, blocked with PBS/2% BSA/1% Tween20 for 1 h. Alexa fluor488-conjugated anti-MYHC4 (53-6503-82, Invitrogen, Carlsbad, USA) was added at 1:100 dilution for 24 h at 4 °C. For phosphorylated S10, histone H3 staining rabbit polyclonal anti-Histone H3 (phospho S10) antibody (ab5176, Abcam, Cambridge, UK) was added at 1:4000 dilution for 1 h at room temperature. After washing three times with PBS, cells were labeled with MACH 2 Anti-Rabbit HRP-Secondary Polymer solution (RHRP520, Biocare Medical, Pacheco, USA) and counterstained with DAPI. Pictures were taken on a fluorescent microscope (FLoid™ Cell Imaging Station).

### C2C12 cell culture and differentiation

Murine myoblast C2C12 cell line was obtained from ATCC (CRL-1772) and cells were maintained according to the company’s instructions. In brief, cells were cultured in DMEM supplemented with 10% FBS, 100 U/ml penicillin, and 100 μg/ml streptomycin (growth medium) at 37 °C in 5% CO2 and 95% air at 100% humidity. The absence of mycoplasma was tested using PCR Mycoplasma Test Kit I/C (PromoCell, Heidelberg, Germany). For gene expression analysis cells were plated into 24-well plates, while for immunofluorescent staining into 96-well plates at a density of 3500 cells/cm^2^. For the 6-day differentiation period, DMEM medium containing 2% FBS and 1% ITS (insulin, transferrin, and sodium selenite) (low serum differentiation medium) was used and replaced every 2^nd^ day with fresh one. In some cases, 1 μM BMS-777607 was added to the wells. To evaluate myoblast fusion, cells were stained with MYHC4 antibody and DAPI, as described previously. Digitally captured photos were taken and analyzed using ImageJ software. The fusion index was calculated by expressing the number of nuclei within MYHC4-positive myotubes with ≥3 nuclei as percentage of the total nuclei (*n* = 500), additionally the length of fibers was measured. Viable cell number was assessed using PrestoBlue (ThermoFisher, Waltham, USA) staining according to the manufacturer’s instructions. Fluorescence was measured on Synergy^TM^ H1 microplate reader. Dying cells in culture were labeled with propidium iodide (80 μg/ml) for 5 min, while total cell number was determined by DAPI staining.

### Gene expression analysis

RNA from magnetically separated muscle-derived F40/80^+^, CD45^+^ and C2C12 cells, and total TA muscles, was isolated with TRIzol (Invitrogen, Carlsbad, USA) reagent according to the manufacturer’s instructions. Mer^+/+^and ^−/−^ control and regenerating TA muscles were homogenized in TRIzol using a Shakeman homogenizer (BioMedical Science, USA). Total RNA was isolated by using the TRI reagent according to the manufacturer’s guidelines (ThermoFisher, Waltham, MA, USA). Total RNA was reverse-transcribed into cDNA using a High Capacity cDNA Reverse Transcription Kit (Life Technologies, Budapest, Hungary) according to the manufacturer’s instruction. qRT-PCR was carried out in triplicates using predesigned FAM-labeled MGB assays (Life Technologies, Budapest, Hungary), including LightCycler 480 Multiwell 384 white plates sealed with adhesive tapes on a Roche LightCycler LC 480 real-time PCR instrument. Relative mRNA levels were calculated using the comparative CT method and were normalized to β-actin mRNA. In case of the total muscle samples, gene expressions were normalized to the total RNA content (200 ng) of the samples. Catalog numbers of the TaqMan assays used were the following: Actb Mm02619580_g1, Itgb1 Mm01253230_m1, Tgfb1 Mm01178820_m1, Myod1 Mm00440387_m1, Myhc1 Mm01332489_m1, Myog Mm00446194_m1, Tnf Mm00443258_m1, Gdf3 Mm00433563_m1, IL1B Mm00434228_m1, IL10 Mm01288386_m1, IL6 Mm00446190_m1, Arg1 Mm00475988_m1, Mer Mm00434920_m1, Axl Mm00437221_m1, Tyro3 Mm00444547_m1, Pax7 Mm00834082_m1.

### Western blot analysis

For detecting MYHC4, Mer, Tyro3, or Axl protein expression in the differentiating C2C12 cells or in wild-type muscles, the whole-cell homogenate was used. The homogenates were prepared in ice-cold lysis buffer (10% v/v glycerol, 1% v/v Triton X-100, 1 mM EGTA, 20 mM Tris, pH 7.9, 100 μM β-glycerophosphate, 137 mM NaCl, 5 mM EDTA, 1.04 mM AEBSF, 0.8 μM aprotinin, 40 μM bestatin, 14 μM E-64, 20 μM leupeptin, and 15 μM pepstatin A). The protein content of the samples was determined by Bio-Rad Protein Assay Dye (Bio-Rad, Budapest, Hungary), and then the homogenate was boiled in a loading buffer with an aliquot corresponding to 40 μg of protein. Proteins were run on a polyacrylamide gel and blotted onto polyvinylidene difluoride membranes using the Bio-Rad electrophoresis and transfer system. Proteins were visualized by anti-MYHC4 (cat#: 53-6503-82), anti-Mer (cat#: 16-5751-85), anti Tyro3 (cat#: PA5-14737), or anti-Axl (cat#: PA5-106118) (all from Invitrogen, Carlsbad, USA) antibodies. Equal loading of proteins was demonstrated by probing the membranes with anti-α tubulin (sc-5286, Santa Cruz Biotechnology, Dallas, USA) antibodies.

### In vitro phagocytosis assay

Phagocytosis assay was performed as described previously [32]. Briefly, target C2C12 cell necrosis was induced by heating the cells for 10 minutes at 65 °C. C2C12 cells were stained with 1 µM CellTracker Deep Red Dye (ThermoFisher, Waltham, USA) and added to MΦs at 5:1 ratio (dead cell/MΦ). After 1-h co-culture, target cells were washed away extensively and MΦs were detached by EDTA. MΦs were labeled with Alexa Fluor 488-conjugated anti-F4/80 antibody (Invitrogen, Carlsbad, USA) for 20 min and the percentage of engulfing cells was determined on a Becton Dickinson FACSCalibur flow cytometer.

### Quantification of necrotic areas

Areas of necrosis were identified based on the following histological criteria: blurring of cell borders, cytoplasmic fragmentation, caliber variation, cell distances, loss of nuclei, and increased immune cell infiltration. Necrotic myofibers were defined as pink pale patchy fibers that are infiltrated by basophil single cells and quantified as described previously [33]. Briefly, 4 nonoverlapping microscope view field areas were digitally captured from 6–8 H&E-stained TA muscle sections at 200-fold magnification. The percentage of necrotic area/total regenerating area was calculated after the manual outlining the necrotic fibers in the sections.

### Statistical analysis

All the data are representative of at least three independent experiments and all data are expressed as mean or median±SEM. Statistical analysis was performed using two-tailed, unpaired Student’s *t*-test and ANOVA with post hoc Tukey HSD test. The equal variance of the samples was tested by *F*-test. * indicates *p* < 0.05, ** indicates *p* < 0.01.

## Results

### Mer deficiency impairs TA muscle regeneration

**Fig. 1:**
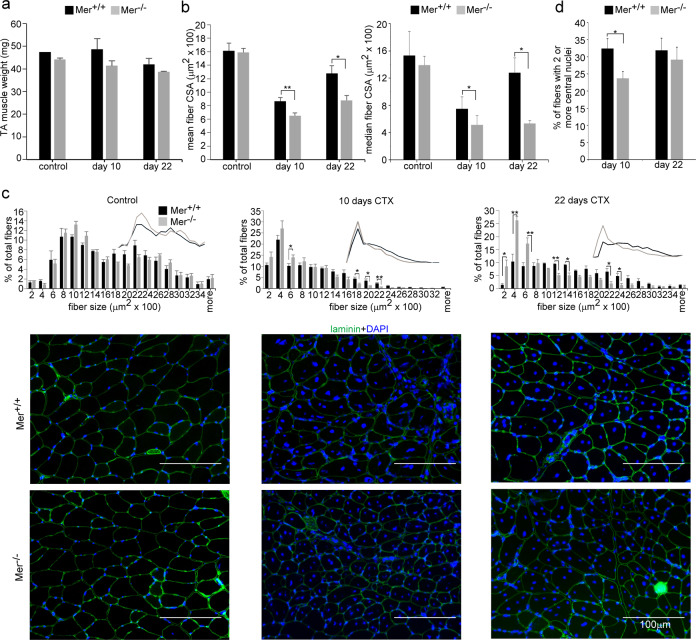
Muscle regeneration is impaired in Mer^−/−^ mice. Muscle injury was induced by injecting 50 μl of 12 μM cardiotoxin (CTX) into the tibialis anterior (TA) muscle of Mer^+/+^ and Mer^−/−^ mice. **a** Muscle weights, **b** mean and median myofiber cross-sectional areas (CSA), and **c** distribution of myofiber sizes in control TA muscles of Mer^+/+^ and Mer^−/−^ male mice, and at day 10 and 22 post-CTX–induced injury together with their representative immunofluorescence images of laminin (green) and DAPI (blue) nuclear staining. Scale bars, 100 µm. In total, 500 or more myofibers were analyzed in each sample using ImageJ software. Data are expressed as mean or median ± SEM. **d** Percentage of newly formed myofibers containing two or more central nuclei in TA muscles of Mer^+/+^ and Mer^−/−^ mice at day 10 and 22 post-CTX–induced injury. Data are expressed as mean ± SEM (*n* = 6). Asterisks indicate statistical significance (*P < 0.05, Student’s *t*-test).

To study a possible role of Mer in muscle homeostasis and regeneration, we compared the muscle weights and the myofiber CSAs of vehicle- and CTX- treated TA muscles from Mer^+/+^ and Mer^−/−^ mice. There was no significant difference between the body weight of Mer^+/+^ and Mer^−/−^ mice (data not shown). TA muscle weights were also not different between control and regenerating muscles at day 10 and 22 post injury in Mer^−/−^ mice as compared to the wild-type controls (Fig. 1a).

There was also no difference in the fiber size between Mer^+/+^ and Mer^−/−^ muscles before injury, but the mean and median CSA of newly formed myofibers with central nuclei in Mer^−/−^ mice were significantly smaller than in Mer^+/+^ mice at day 10 and 22 post injury (Fig. 1b). The CSA frequency distribution showed similar fiber size distribution in control Mer^+/+^ and ^−/−^ mice, but the frequency of smaller fibers was higher, while that of bigger fibers was lower in regenerating Mer^−/−^ muscles as compared to wild type ones (Fig. 1c).

The number of myofibers with two or more central nuclei is an indicator of myoblast fusion in the regenerating muscles. The number of newly formed fibers with two or more central nuclei was decreased in Mer^−/−^ mice as compared to wild type mice at day 10 post injury, but this difference disappeared by day 22 post injury (Fig. 1d). These preliminary data indicated a delay in the skeletal muscle regeneration in the absence of Mer.

Since previous studies indicated that female hormones affect the muscle regeneration process [34], we repeated these experiments with female mice as well (Fig. S1). We could confirm generally a smaller muscle cross-sectional area in female muscles, but found similar alterations in the Mer null female muscle regeneration that we observed in males.

### Delayed differentiation of satellite cells after injury in the absence of Mer

To assess the involvement of Mer in muscle regeneration, the mRNA levels of TAM kinases, that of myogenic genes, such as Pax7 and the MyoD transcription factors involved in SC proliferation and differentiation, as well as that of the myosin heavy chain 1 (MYHC1), a myoblast differentiation marker, were examined in the control and regenerating TA muscles. Additionally, the protein expression levels of the skeletal muscle Mer, Axl, and Tyro3 were also determined.

**Fig. 2:**
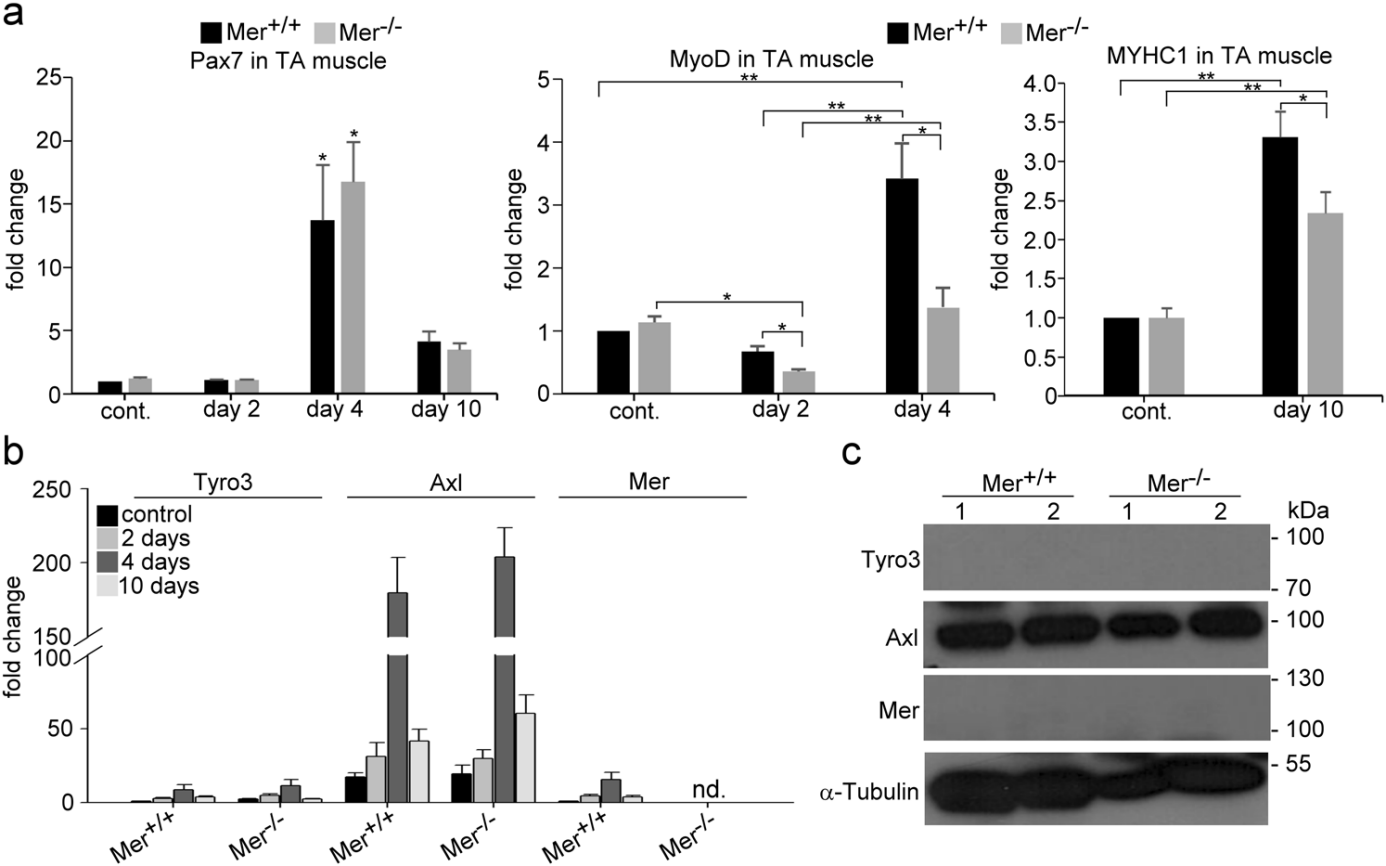
Expression of TAM kinase family members and myogenic genes in the TA muscle of wild type and Mer null mice. **a** mRNA expressions of myogenic marker genes Pax7, MyoD and MYHC1 in control and in regenerating wild type and Mer null TA muscles determined by qRT-PCR at day 2, 4, or 10 post-CTX–induced injury. **b** mRNA expression levels of Mer, Axl and Tyro3 in control and in regenerating wild type and Mer null TA muscles determined by qRT-PCR at day 2, 4, or 10 post-CTX–induced injury. **c** Protein levels of Mer, Axl, and Tyro3 in the TA muscle of wild-type and Mer null mice determined by Western blot analysis. α Τubulin was used as a loading control. Data are expressed as mean ± SEM (*n* = 3). Asterisks indicate statistical significance (*P < 0.05, **P < 0.01, ANOVA test).

The mRNA levels of Pax7, the SC-specific transcription factor, were strongly induced by day 4, and loss of Mer did not affect this induction (Fig. 2a). However, the expression of MyoD was lower in the muscles of Mer^−/−^ mice as compared to wild-type ones at days 2 and 4 after CTX-induced muscle injury, while that of MYHC1 was lower in Mer^−/−^ mice compared with Mer^+/+^ mice at day 10 post injury indicating a delayed differentiation (Fig. 2a). Among the TAM kinases Axl mRNA was dominantly expressed by the skeletal muscle, and its expressions showed a similar pattern to that of Pax7 during muscle regeneration (Fig. 2b). Similar to Pax7, loss of Mer did not affect the expression of Axl either. In accordance with the mRNA data, we could detect only the expression of Axl on protein level in the mouse skeletal muscle (Fig. 2c) similar to human studies, which also demonstrated that Axl is the dominant TAM kinase expressed by skeletal muscle cells (https://www.proteinatlas.org). These findings indicate that not the SCs or myoblasts are the cells, where loss of Mer might primarily affect muscle regeneration.

### Normal recruitment of MΦs and neutrophils after injury in the absence of Mer

**Fig. 3:**
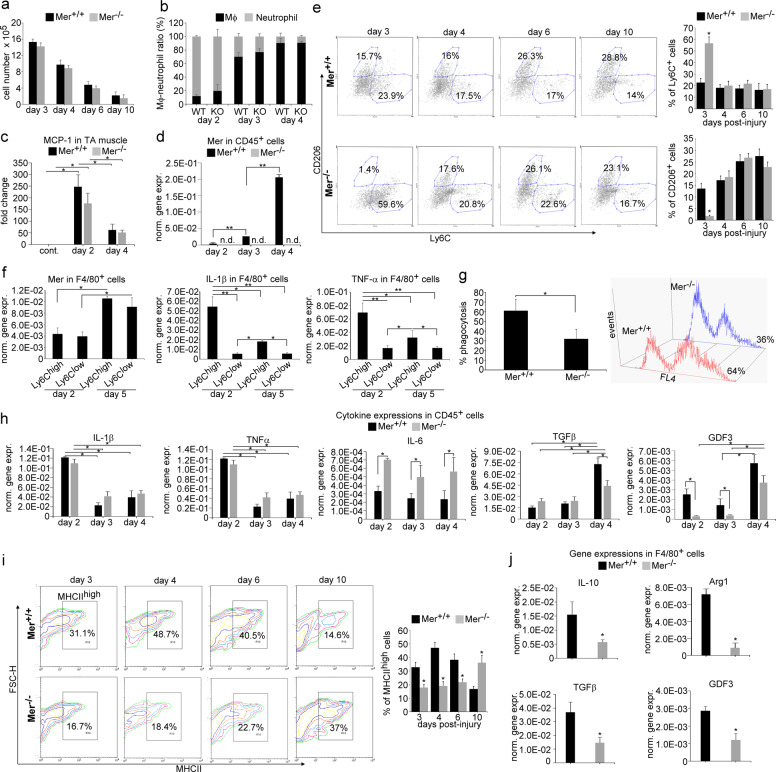
As compared to wild-type mice, leukocyte infiltration is not altered in Mer null regenerating TA muscles following cardiotoxin injury, but the pro-inflammatory to healing phenotypic conversion of macrophages is delayed. Alterations **a** in the number of CD45^+^ leukocytes per injured muscle, **b** ratio of anti-F4/80 antibody-stained MΦs and anti-Ly6G/Ly6C- (GR-1) stained neutrophils within the CD45^+^ leukocyte population in Mer^+/+^ and Mer^−/−^ TA muscles during the first 4 days of regeneration following CTX-induced injury (*n* = 3). **c** MCP-1 mRNA expression levels of muscle-derived wild type or Mer null CD45^+^ leukocytes determined by qRT-PCR following CTX-induced injury (*n* = 3). **d** Changes in Mer mRNA expressions of CD45^+^ infiltrating leukocytes isolated from Mer^+/+^ and Mer^−/−^ regenerating TA muscles following CTX injury determined by qRT-PCR at the indicated time points (*n* = 6). **e** Representative scatter plots of CD206- and Ly6C-stained muscle-derived F4/80^+^ cells determined at the indicated days following CTX-induced injury (*n* = 3). **f** Mer, IL-1β, and TNF-α mRNA expressions in Ly6C^high^ and Ly6C^low^ F4/80^+^ cells isolated from TA muscles determined by qRT-PCR at days 2 and 5 post injury (*n* = 3). **g** Necrotic C2C12 myoblast cell phagocytic capacity of muscle-derived Mer^+/+^ and Mer^−/−^ MΦs (MMΦ) isolated at day 4 post-CTX-induced injury determined by flow cytometric analysis (*n* = 3). **h** Changes in the mRNA expressions of the indicated cytokines in CD45^+^ infiltrating leukocytes isolated from Mer^+/+^ and Mer^−/−^ regenerating TA muscles following CTX-induced injury determined by qRT-PCR (*n* = 6). **i** Representative scatter plots of MHCII-stained muscle-derived F4/80^+^ cells determined at the indicated days following CTX-induced injury (*n* = 3). **j** mRNA expressions of the indicated M2 phenotypic markers in Mer^+/+^ and in Mer^−/−^ muscle-derived MΦs isolated at day 4 post CTX-induced injury determined by qRT-PCR (*n* = 6). All data are expressed as mean ± SEM. Asterisks indicate statistical significance (*P < 0.05, **P < 0.01, Student’s *t*-test and ANOVA test).

Since Mer is involved in the phagocytosis of both apoptotic and necrotic cells by macrophages [32], and altered efferocytosis might affect the muscle regeneration program [10], our interest turned to the inflammatory cells. Migration of inflammatory cells to the injured area and tissue inflammation plays a crucial role in the muscle regeneration process following injury. To assess the composition of leukocytes in the early phase of muscle regeneration, we performed flow cytometric analysis of magnetically separated CD45^+^ cells from collagenase-digested muscles (Fig. S2a). In accordance with previous observations, we detected early infiltration of neutrophils at day 2 post injury followed by increasing numbers of MΦs at days 3 and 4 in wild-type mice. Loss of Mer did not affect the number of infiltrating CD45^+^ cells (Fig. 3a), the neutrophil/MΦ ratios (Fig. 3b), or the level of monocyte chemoattractant protein-1 (MCP-1), the main chemoattractant signal for MΦ recruitment [35] (Fig. 3c) in the regenerating muscle.

### Decreased phagocytic capacity of MΦs and lower IL-10 and GDF3 expression in muscle-derived CD45^+^ leukocytes in the absence of Mer

We also determined the mRNA expression of Mer and various cytokines in the CD45^+^ cells. We found that early infiltrating wild-type CD45^+^ cells expressed Mer, and the M2- specific Mer expression was significantly induced by day 4 (Fig. 3d) in accordance with the formation of the M2-like Ly6C^low^ healing MΦs on this day (Fig. 3e). Surprisingly, however, when we separated the Ly6C^high^ and the Ly6C^low^ F4/80^+^ MΦs (Fig. S2b), we found that Mer mRNA expression was induced with time in both populations (Fig. 3f), and the expression of it did not show a correlation with the Ly6C expression, despite the fact that the pro-inflammatory IL-1β, or TNF-α productions were associated with the Ly6C^high^ pro-inflammatory population (Fig. 3f), as reported [6–9]. Efferocytosis of cell debris followed by a switch from a pro-inflammatory to an anti-inflammatory environment plays a key role during skeletal muscle regeneration. Fortifying the importance of Mer in dead cell engulfment, Mer^−/−^ muscle-derived F4/80^+^ MΦs isolated at day 4 post injury displayed significantly lower necrotic myoblast phagocytic capacity than the wild-type ones (Fig. 3g).

During muscle regeneration the M1-specific IL-1β, TNF-α, and IL-6 expression decreased, while the M2-specific TGFβ and GDF3 expression of CD45^+^ leukocytes increased in time in both strains. However, we found a significantly higher expression of IL-6, and significantly lower M2-specific TGFβ and GDF3 expression in the Mer^−/−^ CD45^+^ leukocytes, as compared to the wild-type ones (Fig. 3h).

### Delayed pro-inflammatory/healing phenotypic switch in Mer null macrophages during the muscle regeneration process

Since the data above suggested that Mer null MΦs might have an altered pro-inflammatory/healing phenotypic switch during muscle regeneration, we followed the phenotypic changes of MΦs in both Mer^+/+^ and Mer^−/−^ regenerating muscles. As seen in Fig. 3e, detected at day 3, there was a delay in the disappearance of M1-specific [6–9] Ly6C^high^ and in the appearance of M2-specific CD206 phagocytic receptor [36] expressing F4/80^+^ MΦs in Mer^−/−^ muscles. However, this difference disappeared by day 4. Similarly, a significant delay was observed in the appearance of MHCII^high^ expressing Mer^-/-^ F4/80^+^ MΦs [37] as well (Fig. 3i). In addition, when F4/80^+^ MΦs were separated from regenerating muscles at day 4 post injury and analyzed for various M2 marker expressions, all the checked M2 markers were expressed at a lower amount by Mer null cells (Fig. 3j).

### Mer^−/−^ TA muscles display delayed tissue repair and enhanced collagen deposition

**Fig. 4:**
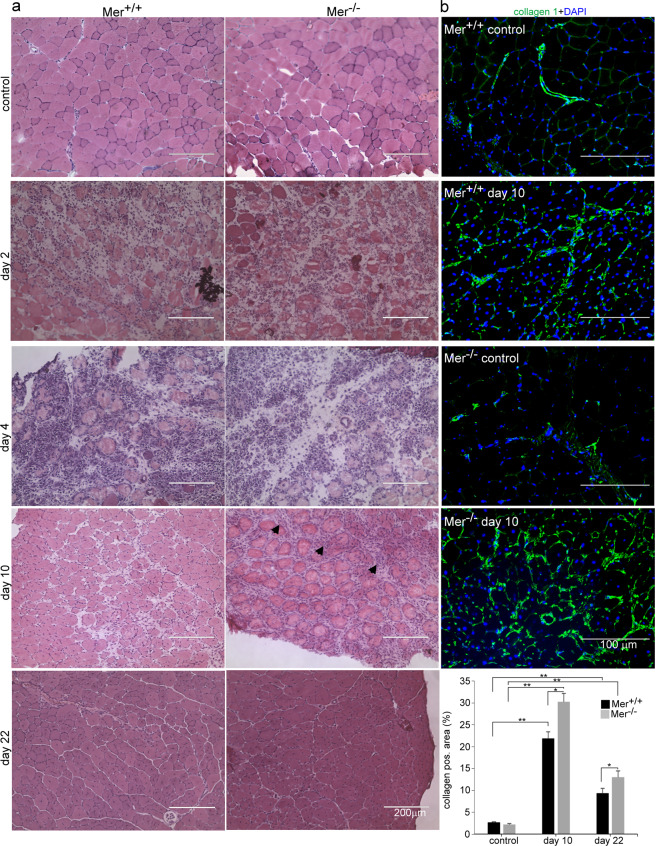
Time-dependent histological morphology and collagen deposition of TA muscles following cardiotoxin-induced injury in Mer^+/+^ and Mer^−/−^ mice. **a** Representative H&E stained cross-sections of Mer^+/+^ and Mer^−/−^ TA muscles without treatment and at 2, 4, 10 and 22 post CTX-induced injury (*n* = 4, except at day 10, when *n* = 6). Arrows indicate necrotic areas. Scale bars, 200 μm. **b** Representative immunofluorescence images of type 1 collagen (green) and DAPI (blue) nuclear staining in control and in Mer^+/+^ and Mer^−/−^ TA muscles regenerating for 10 days, and quantification of collagen 1-positive areas in the control and in the regenerating muscles of Mer^+/+^ and Mer^−/−^ mice at days 10 and 22 post CTX-induced injury. All data are expressed as mean ± SEM (*n* = 4 in controls and *n* = 6 in the CTX-treated muscles). Asterisks indicate statistical significance (*P < 0.05, **P < 0.01, ANOVA test).

Since our data indicated impaired efferocytosis leading to delayed phenotypic switch of muscle MΦs, we thought to determine also the rate of in vivo clearance by comparing the disappearance of necrotic fibers in wild-type and Mer^−/−^ muscles following CTX injection (Fig. 4a). As compared to the control tissue sections, during the first 2 days of injury, Mer^+/+^ and Mer^−/−^ muscles displayed local necrosis and abundant inflammatory cell infiltration. At day 4 post injection, still large numbers of leukocytes and necrotic muscle fibers were visible in the injured muscles of both mouse strains. However, by day 10, in wild-type muscles, most of the necrotic fibers were cleared, while Mer^−/−^ muscles still contained significant amounts of necrotic areas at this time point (Fig. 4a). The percentages of necrotic areas were 7.33 + −2.65 and 18.22 + −3.86 in Mer^+/+^ and Mer^−/−^ muscles, respectively (*P* < 0.01, *n* = 3). However, at day 22 post injury, the overall histological architecture of both Mer^+/+^ and Mer^−/−^ muscles was restored, and necrotic fibers were no longer visible.

In addition to inflammatory macrophages and SCs, efficient muscle repair also requires the migration and proliferation of fibroblasts in order to produce new temporary extracellular matrix components. These elements serve to stabilize the tissue and act as a scaffold for the new fibers. In accordance, we detected an increased amount of collagen I in the regenerating muscles of both Mer^+/+^ and Mer^−/−^ mice as compared to their own non-regenerating muscles with a significantly higher collagen deposition in the case of Mer^−/−^ muscles at both days 10 and 22 post-injury (Fig. 4b).

### In vivo inhibition of TAM tyrosine kinase receptors impairs muscle regeneration

**Fig. 5:**
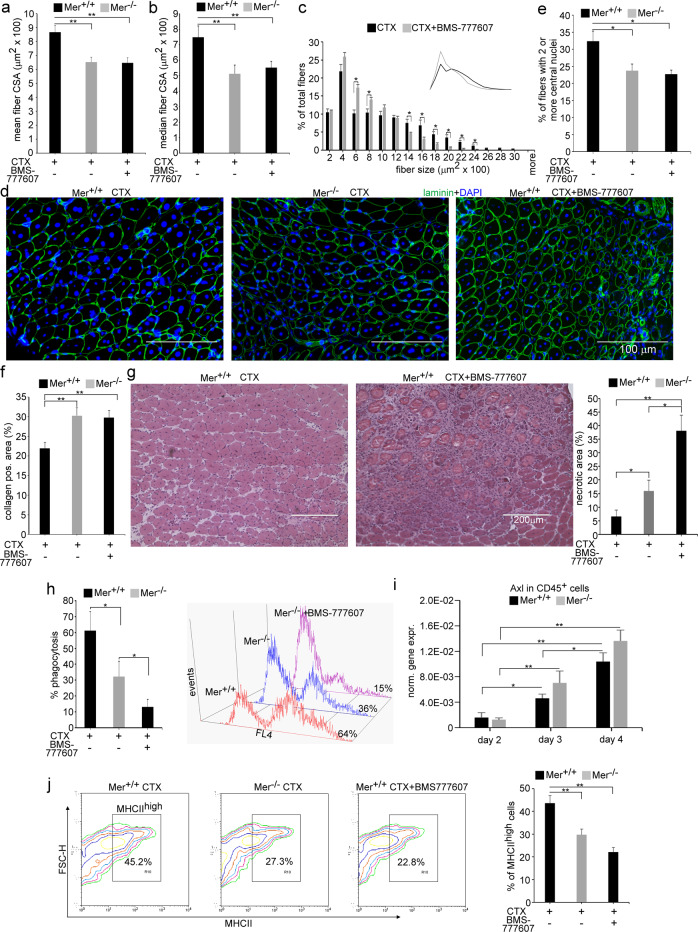
In vivo administration of the pan-TAM tyrosine kinase inhibitor BMS-777607 into wild-type mice added during the early stage of regeneration impairs muscle repair following CTX-induced injury. Muscle injury was induced by CTX as above in the tibialis anterior (TA) muscle of Mer^+/+^ and Mer^−/−^ mice. BMS-777607 was also injected on days 1 and 3 following CTX injection into some Mer^+/+^ mice. TA muscles from the three types of mice were analyzed at day 10 post-CTX-induced injury. **a** Mean and **b** median myofiber cross-sectional areas of BMS-777607-treated Mer^+/+^ muscles in comparison to that found in the Mer^+/+^ and Mer^−/−^ muscles, **c** distribution of myofiber sizes in Mer^+/+^ TA muscles exposed or not to BMS-777607, and **d** representative immunofluorescence images of laminin (green) and DAPI (blue) nuclear staining of the three types of CTX-treated muscle. Scale bars, 200μm. In total, 500 or more myofibers were analyzed in each sample using ImageJ software (*n* = 6). **e** Percentage of newly formed myofibers containing two or more central nuclei in regenerating Mer^+/+^ muscles exposed or not to BMS-777607 in comparison to that found in the Mer^−/−^ muscles (*n* = 6). **f** Quantification of the type 1 collagen-positive areas in regenerating Mer^+/+^ muscles exposed or not to BMS-777607in comparison to that found in the Mer^−/−^ muscles (*n* = 4). **g** Representative H&E-stained sections from Mer^+/+^ muscles regenerating in the presence and absence of BMS-777607 and quantification of their necrotic areas in comparison to that found in the Mer^−/−^ muscles (*n* = 4). Scale bars, 100μm. **h** Necrotic C2C12 myoblast cell phagocytic capacity of muscle-derived Mer^+/+^, Mer^-/-^, and 1 μM BMS-777607-treated Mer^−/−^ MΦs isolated at day 4 post-CTX-induced injury determined by flow cytometric analysis (*n* = 3). **i** Changes in Axl mRNA expressions of CD45^+^ infiltrating leukocytes isolated from Mer^+/+^ and Mer^−/−^ regenerating TA muscles following CTX injury determined by qRT-PCR at the indicated time points (*n* = 6). **j** Representative scatter plots and quantification of MHCII stained F4/80^+^ cells derived from the three types of muscle determined at day 4 following CTX-induced injury (*n* = 3). All data are expressed as mean or median ± SEM. Asterisks indicate statistical significance (*P < 0.05, **P < 0.01, Student’s *t*-test and ANOVA test).

To confirm that the observed impaired muscle regeneration in the Mer-knockout mice is indeed the consequence of a decreased TAM signaling in the muscle MΦs, and not the consequences of an off-target mutation in the knockout strain, we injected the pan-TAM tyrosine kinase inhibitor BMS-777607 into wild-type mice during the first 5 days of CTX -induced injury (Fig. 5) and checked the regeneration process 5 days later at day10 post injury. In harmony with the findings in Mer null mice, we found that the mean and median CSA of newly formed fibers was significantly lower, the frequency of smaller fibers was higher, while that of bigger fibers was lower in regenerating muscles of BMS-777607-treated Mer^+/+^ mice as compared to vehicle-treated ones (Fig. 5a–d). The number of newly formed fibers with two or more central nuclei was also decreased in the BMS-777607-treated Mer^+/+^ regenerating muscles as compared to that of the vehicle-treated ones at day 10 post injury (Fig. 5e). Similarly, the collagen deposition (Fig. 5f) and necrosis (Fig. 5g) were also significantly higher in the inhibitor-treated mice as compared to vehicle-treated ones. What is more, none of the results were significantly different, when compared to that of the Mer null mice, with the exception of the size of the necrotic area. This later was significantly larger in the inhibitor-treated mice indicating delayed clearance as compared to the Mer null mice. Accordingly, inhibition of TAM receptor kinases by BMS-777607 further inhibited efferocytosis by muscle Mer^−/−^ MΦs (Fig. 5h). Since Tyro3 is not significantly expressed by MΦs [38], we determined the mRNA expression of Axl in the CD45^+^leukocyte population in which only macrophages express Axl. As shown in Fig. 5i, Axl was indeed expressed by CD45^+^leukocytes, but its expression only slightly increased with time, as the macrophages started dominating the CD45^+^leukocyte population. Addition of BMS-777607 also induced a delay in the macrophage phenotypic switch of MΦs, but not more than the loss of Mer alone demonstrated by the delayed appearance of MHCII^high^ cells (Fig. 5j).

Similar experiments by injecting BMS-777607 on the fifth, seventh and ninth days did not affect the muscle regeneration process detected at day 10 post injury (Fig. S3b). These observations indicate that Mer and possibly Axl are required during the first days of the skeletal muscle regeneration process. Since loss of Mer results in a delay of the phenotypic change of macrophages leading to a delayed muscle regeneration, but additional inhibition of Axl by BMS-777607 did not induce a further delay in the regeneration, our data suggest that though Axl might contribute to the clearance by MΦs, Mer drives dominantly the inflammatory to repair phenotype transition of macrophages and consequently the release of those macrophage-derived growth factors that drive myogenesis. In addition, because Axl is also expressed by muscle cells, we could not exclude that BMS-777607 could also affect the early proliferating and differentiating myoblasts contributing to the larger necrotic areas observed at day 10 of regeneration in inhibitor-treated muscles.

### Axl is a myotube growth and survival receptor for the C2C12 myoblasts

**Fig. 6:**
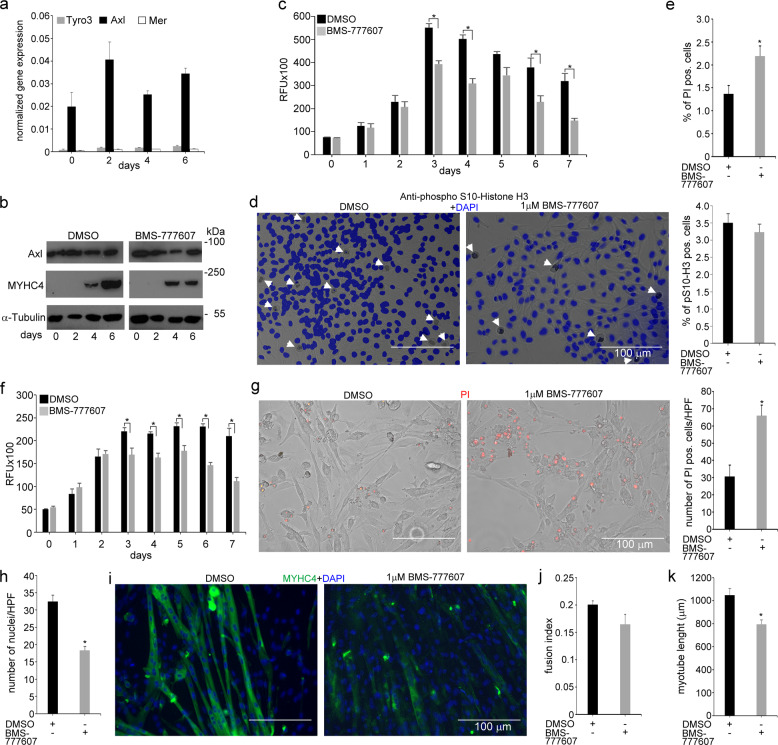
In vitro administration of the pan-TAM tyrosine kinase inhibitor BMS-777607 impairs myogenesis of C2C12 myoblast cells. **a** mRNA expression levels of Tyro3, Axl, and Mer during the differentiation of C2C12 myoblast cells determined by qRT-PCR. **b** Protein expression levels of Axl and myosin heavy chain 4 (MYHC4) in differentiating C2C12 myoblasts in the presence or absence of 1 μM BMS-777607 determined by Western blot analysis. α-Tubulin was used as a loading control. One representative blot of three is shown. **c** Alterations in the number of viable C2C12 cells grown in growth medium in the presence or absence of 1 μM BMS-777607 determined by PrestoBlue staining. **d** Percent of cells in G2/M phase grown in growth medium in the presence or absence of 1 μM BMS-777607 as indicated by anti-phospho-histone H3 (Ser10) and DAPI co-staining (at least 20 HPF were analyzed). Arrows point to the anti-phospho-histone H3 positive nuclei. **e** Percent of PI-positive cells grown in growth medium in the presence or absence of 1 μM BMS-777607. **f** Alterations in the number of viable C2C12 cells grown in differentiation medium in the presence or absence of 1 μM BMS-777607 determined by PrestoBlue staining. BMS-777607 was added when the medium was changed to differentiation medium. **g** Representative light microscopic images of C2C12 myoblasts differentiated for 6 days in the absence or presence of 1 μM BMS-777607 after staining dead cells with propidium iodide and quantification of propidium iodide-positive nuclei per high-power field (at least 20 HPF were analyzed). **h** Number of nuclei per high-power field of C2C12 cells differentiated for 6 days in the absence or presence of 1 μM BMS-777607. **i** Representative fluorescent microscopic images of C2C12 myoblasts differentiated for 6 days in the absence or presence of 1 μM BMS-777607. MYHC4 was visualized by using anti-MYHC4 antibody (green) and nuclei by DAPI (blue). **j** Fusion index of C2C12 cells differentiated for 6 days in the absence or presence of 1 μM BMS-777607. **k** The length of myotubes generated from C2C12 myoblasts differentiated for 6 days in the absence or presence of 1 μM BMS-777607. All the data are expressed as mean ± SEM of three independent experiments. Asterisks indicate statistical significance (*P < 0.05, Student’s *t*-test). Scale bars, 100 µm.

Shifting mouse C2C12 myoblasts from growth medium to low-serum fusion medium induces formation of multinucleated, myosin-expressing myotubes [39], and provides a quantifiable in vitro model of myogenesis [40]. We used this model to study whether TAM receptor signaling is required for the myogenesis process. Since skeletal muscle cells do not express Mer or Tyro3, we determined first the mRNA (Fig. 6a) and protein expression (Fig. 6b) levels of the three TAM kinases in the C2C12 myoblasts as well. Similar to the in vivo data, we could not detect protein expressions of Mer or Tyro3 (data not shown). Axl, however, was expressed by C2C12 myoblasts (Fig. 6a and b) and its expression level did not alter during their differentiation. Exposure of C2C12 myoblasts to BMS-777607 in growth medium led to a slower cell growth rate by the third day (Fig. 6c). The reduced growth rate, however, was not the result of a reduced cell proliferation detected by the percentage of pS10 histone H3-positive cells (Fig. 6d), rather that of an increased rate of cell death (Fig. 6e). Shifting C2C12 cells to the low-serum fusion medium also resulted in an inhibition of the cell growth rate by the third day, but it was more pronounced in the inhibitor-exposed cells (Fig. 6f). The number of myoblasts did not change during the following days of differentiation in any of the cultures, but on day 6, the number of inhibitor-treated cells started to decrease. Accordingly, on day 6, the number of propidium iodide-labeled dead cells floating in the culture medium increased significantly in the inhibitor-treated culture (Fig. 6g), while that of the DAPI-labeled nuclei per high-power field decreased (Fig. 6h, i).

Exposure of differentiating myoblasts to BMS-777607 did not affect the expression of Axl. Neither was the differentiation of C2C12 myoblasts detected with the help of the MYHC4 expression affected until day 6, when the expression of MYHC4 in the inhibitor-treated cultures did not increase further (Fig. 6b). BMS-777607 did not interfere with the myoblast fusion either (Fig. 6i, j). However, the fused myoblasts showed an impaired growth in the absence of TAM kinase signaling (Fig. 6i, k).

## Discussion

In the present study, the involvement of Mer in the skeletal muscle regeneration process was studied by using the cardiotoxin injury model. Previous studies have already indicated that infiltrating MΦs play a key role in the orchestration of this biological process. Thus, inhibition of the MΦ infiltration in CCR2 null mice [35], impaired efferocytosis due to genetic ablation of scavenger receptor class BI [33], or impaired MΦ phenotypic change into the healing direction in the absence of the nuclear receptor PPARγ [9] or the Nfix transcription factor [41], all severely delayed the regeneration process.

Mer is a member of the TAM receptor tyrosine kinase family. Genetic studies have shown that TAM signaling plays an essential role, especially in the sentinel cells of the immune system, where the principal receptors are Mer and Axl [19]. Though both receptors are involved in efferocytosis, Mer was shown to be expressed dominantly by tissue-resident macrophages or to function in tolerogenic settings, while Axl was found to be expressed by inflammatory macrophages and to function in inflammatory environments [19]. In line with these observations, Axl was expressed in muscle-derived wild-type CD45^+^ cells from the early phase of regeneration. Mer expressions, however, were induced significantly only by day 4, when a high percentage of infiltrating Ly6C^high^ pro-inflammatory macrophages was already converted to Ly6C^low^ healing macrophages. Interestingly, however, Mer expression was independent of Ly6C.

Previous studies have indicated that Mer contributes to maintenance of normal homeostasis in organs by promoting efferocytosis by macrophages [22] and via contributing to the initiation of the anti-inflammatory program following apoptotic cell uptake [23, 24, 26]. Though cytokine production during the early efferocytosis of damaged muscle cells by muscle tissue-resident macrophages expressing Mer very likely plays a role in initiating recruitment of neutrophils and then that of the infiltrating macrophages following muscle injury, we have not found a difference in the production of MCP-1 or in the number or composition of the recruited cells in Mer null muscles.

We found, however, that Mer significantly contributed to the efferocytosis by post-injury day 4 MΦs, though based on the TAM kinase inhibitory experiments, it was not the only TAM kinase receptor to do so. Consistent with this finding, we detected significantly increased necrotic tissue areas in the knockout muscle at day 10 post injury in the TA muscles, but this difference was no longer present at day 22 post-injury highlighting the role of other muscle macrophage phagocytic receptors in the dead cell clearance.

In line with the impaired efferocytosis, we could also demonstrate a delayed pro-inflammatory/healing macrophage conversion in the absence of Mer, as both the disappearance of Ly6C-positive, and the appearance of CD206^+^ or MHCII^high^ macrophage population, were delayed in the Mer^−/−^ regenerating muscle. Simultaneously, we detected significantly reduced CSA in the regenerating muscles of Mer^−/−^ mice. Since Mer is not expressed by the skeletal muscle, the smaller CSA must be the consequence of impaired growth of newly formed fibers and/or of a slower myoblast fusion rate in the muscle as a result of the decreased growth factor production by Mer^−/−^ MΦs. Similar was the finding, when wild-type mice were injected with BMS-777607 that inhibits both Mer and Axl signaling underlying the dominant role of macrophage Mer in this phenotype. In addition, however, we detected much longer persisting larger necrotic areas in the TA muscles of inhibitor-treated mice at day 10 post CTX-induced injury indicating a further reduced ΜΦ efferocytotic capacity in the absence of ΜΦ Axl, and also a possible involvement of the muscle Axl in the myogenesis process.

Axl is known to be activated via the bridging molecule GAS6 [42] and myoblasts release GAS6 to support Axl signaling in an autocrine manner [43]. The mechanism of TAM receptor activation is unique among receptor tyrosine kinase families, requiring both a protein ligand and the lipid moiety PS [44]. When muscle cells die following injury, they can provide this PS for both the engulfing macrophages, as well as for the early proliferating and differentiating SCs, which already express Axl [45]. In addition, increasing evidence indicates that not only phagocytosis of apoptotic cells, but myoblast fusion is also PS-dependent [46]. During myoblast fusion, PS appears at the fusing cell–cell contact areas and almost exclusively only on mononucleated myoblasts in contact with other mononucleated cells or small myotubes containing only a few nuclei [46]. This asymmetric PS location will trigger Axl and provide myotube survival and growth signal always in those myotubes, which undergo continuous fusion. The interaction between PS and two PS-recognizing efferocytosis receptors (brain-specific angiogenesis inhibitor 1 (BAI1) and stabilin-2) on the surface of myoblasts has already been reported to be a prerequisite for cell fusion during muscle fiber formation [38, 47]. Our data identify Axl, as a third PS-dependent efferocytosis receptor to be also required for proper myogenesis, but acting as a myotube growth and survival receptor. The potential involvement of GAS6 and Axl in the muscle development process is also supported by a recent observation that the dominant phenotype of GAS6/Axl double-knock out mice is a significantly reduced skeletal muscle mass [48]. Altogether, our data demonstrate that intact TAM kinase receptor signaling is required for the proper muscle regeneration process both in the muscle-derived macrophages and in the myoblasts.

## Supplementary information

Supplementary figure legends

Supplementary figure 1

Supplementary figure 2

Supplementary figure 3
