# Supplementary material for: TAM kinase signaling is indispensable for proper skeletal muscle regeneration in mice
Source: Cell Death Dis. 2021 Jun 12;12(6):611. doi: 10.1038/s41419-021-03892-5 (PMC8197762; doi:10.1038/s41419-021-03892-5)
Supplement: Supplementary file 1 — Supplementary figure legends [file 41419_2021_3892_MOESM1_ESM.docx]

**Supplementary figure legends**

**Fig. S1: Muscle regeneration is impaired in Mer^-/-^ female mice.** Muscle injury was induced by injecting 50 μl of 12 μM cardiotoxin (CTX) into the tibialis anterior (TA) muscle of Mer^+/+^ and Mer^-/-^ female mice. **a** Muscle weights, **b** mean and median myofiber cross-sectional areas (CSA), and **c** distribution of myofiber sizes in control TA muscles of Mer^+/+^ and Mer^-/-^ female mice, and at day 10 and 22 post-CTX–induced injury together with their representative immunofluorescence images of laminin (green) and DAPI (blue) nuclear staining. Scale bars, 100 µm. 500 or more myofibers were analyzed in each sample using ImageJ software. Data are expressed as mean or median ± SEM (n=6). Asterisks indicate statistical significance (*P<0.05, Student’s t-test).

**Fig. S2: Flow cytometric analysis of CD45^+^ cells isolated from regenerating TA muscles of wild type and Mer null mice.** **a** Representative scatter plots of Ly6C/G and F4/80 stained muscle-derived CD45^+^ cells isolated at the indicated days after CTX-induced injury. **b** Representative scatter plots Ly6C stained Ly6C^high^ and Ly6C^low^ F4/80^+^ cells after cell sorting.

**Fig. S3: Administration of the pan-TAM tyrosine kinase inhibitor BMS-777607 after 5 days post-CTX-induced injury does not affect muscle regeneration.** Muscle injury was induced by CTX as above in the tibialis anterior (TA) muscle of Mer^+/+^ and Mer^-/-^ mice. BMS-777607 was also injected on day 5, 7, and 9 following CTX injection into some Mer^+/+^ mice. TA muscles from the three types of mice were analyzed at day 10 post-CTX-induced injury. **a** Mean and **b** median myofiber cross-sectional areas of BMS-777607-treated Mer^+/+^ muscles in comparison to that found in the Mer^+/+^ and Mer^-/-^ muscles, and **c** distribution of myofiber sizes in Mer^+/+^ TA muscles exposed or not to BMS-777607. 500 or more myofibers were analyzed in each sample using ImageJ software. Data are expressed as mean or median±SEM (n=6 legs). **d** Representative H&E-stained sections from Mer^+/+^ muscles regenerating in the presence and absence of BMS-777607. Scale bars, 200μm. **e** Percentage of newly formed myofibers containing two or more central nuclei in the TA muscles of the three types of mice. Data are expressed as mean ± SEM (n=6). Asterisks indicate statistical significance (*P<0.05, **P<0.01).
