# Supplementary figures and images for: TAM kinase signaling is indispensable for proper skeletal muscle regeneration in mice
Source: Cell Death Dis. 2021 Jun 12;12(6):611. doi: 10.1038/s41419-021-03892-5 (PMC8197762; doi:10.1038/s41419-021-03892-5)

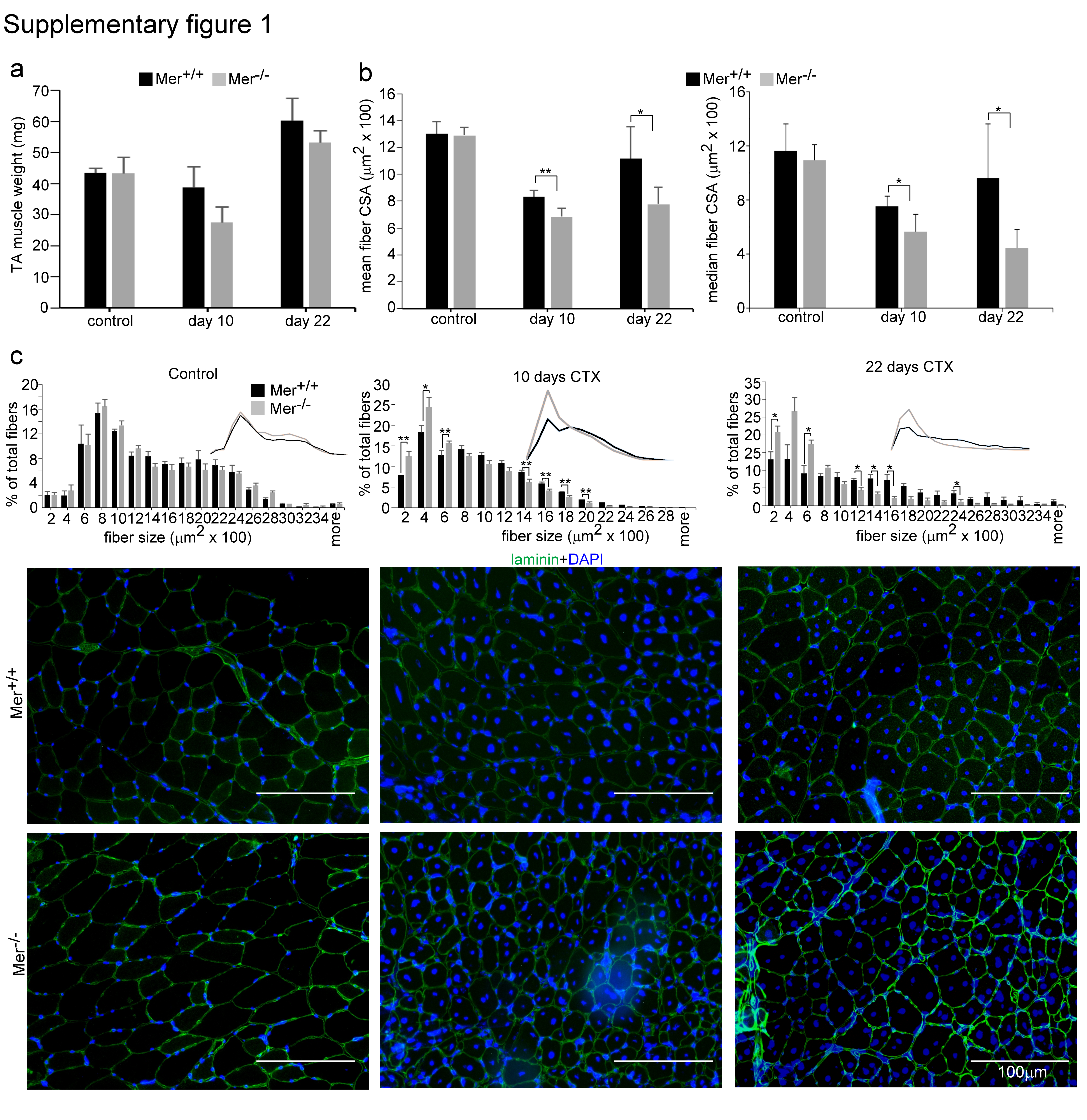

Supplement: Supplementary file 2 — Supplementary figure 1 [file 41419_2021_3892_MOESM2_ESM.tif]

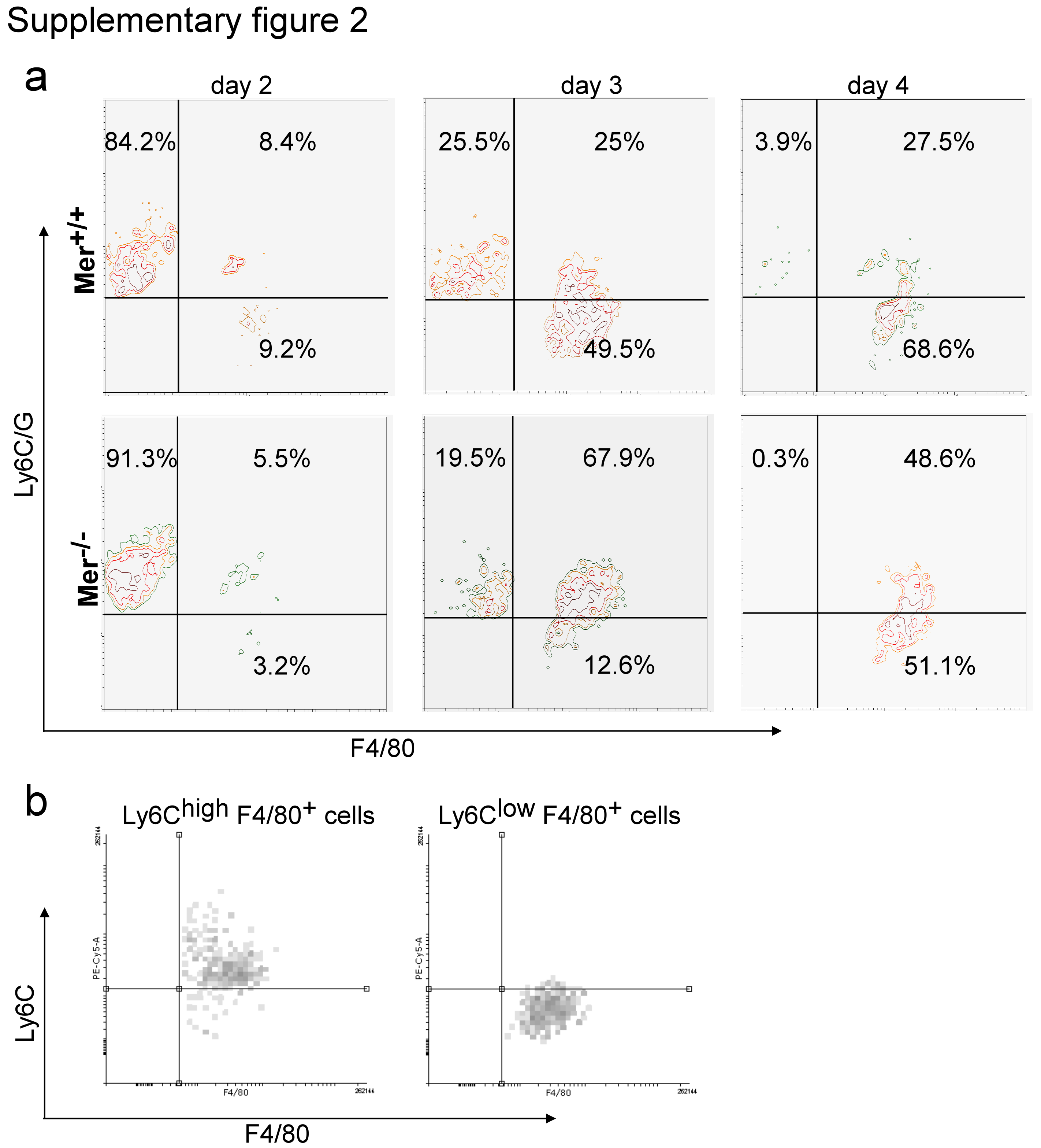

Supplement: Supplementary file 3 — Supplementary figure 2 [file 41419_2021_3892_MOESM3_ESM.tif]

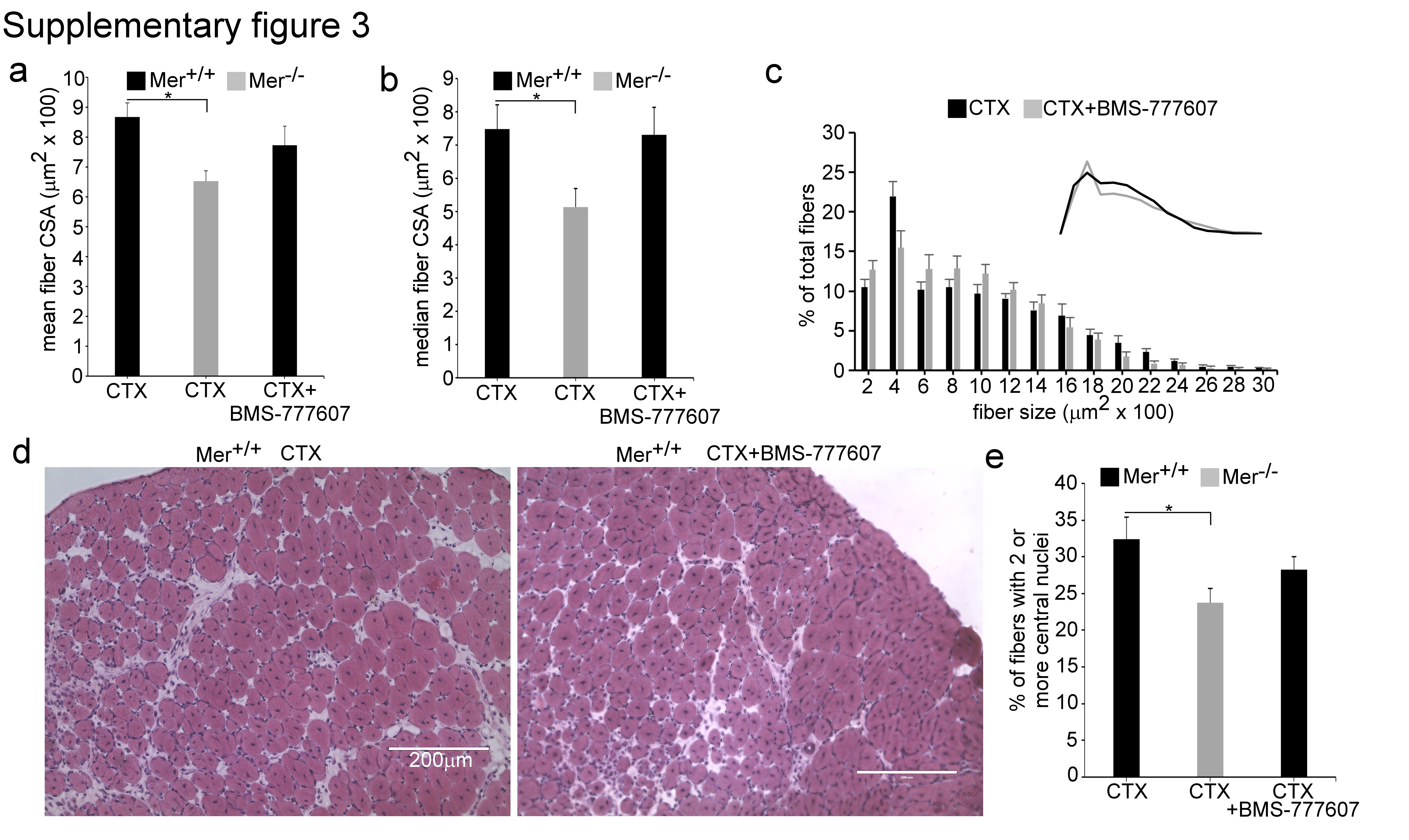

Supplement: Supplementary file 4 — Supplementary figure 3 [file 41419_2021_3892_MOESM4_ESM.tif]
